# Analysis of gene copy number changes in tumor phylogenetics

**DOI:** 10.1186/s13015-016-0088-2

**Published:** 2016-09-22

**Authors:** Jun Zhou, Yu Lin, Vaibhav Rajan, William Hoskins, Bing Feng, Jijun Tang

**Affiliations:** 1 School of Computer Science and Technology, Tianjin University, Tianjin, 300072 China; 2 Department of Computer Science and Engineering, University of South Carolina, Columbia, SC 29208 USA; 3 Research School of Computer Science, Australian National University, Canberra, ACT 0200 Australia; 4 Xerox Research Centre India (XRCI), Bangalore, India

**Keywords:** Tumor phylogeny, Maximum parsimony, Gene copy number, FISH, Rectilinear Steiner minimum tree, Gene duplication, Chromosomal duplication, Whole genome duplication

## Abstract

**Backgound:**

Evolution of cancer cells is characterized by large scale and rapid changes in the chromosomal  landscape. The fluorescence in situ hybridization (FISH) technique provides a way to measure the copy numbers of preselected genes in a group of cells and has been found to be a reliable source of data to model the evolution of tumor cells. Chowdhury et al. (Bioinformatics 29(13):189–98, 23; PLoS Comput Biol 10(7):1003740, 24) recently develop a computational model for tumor progression driven by gains and losses in cell count patterns obtained by FISH probes. Their model aims to find the rectilinear Steiner minimum tree (RSMT) (Chowdhury et al. in Bioinformatics 29(13):189–98, 23) and the duplication Steiner minimum tree (DSMT) (Chowdhury et al. in PLoS Comput Biol 10(7):1003740, 24) that describe the progression of FISH cell count patterns over its branches in a parsimonious manner. Both the RSMT and DSMT problems are NP-hard and heuristics are required to solve the problems efficiently.

**Methods:**

In this paper we propose two approaches to solve the RSMT problem, one inspired by iterative methods to address the “small phylogeny” problem (Sankoff et al. in J Mol Evol 7(2):133–49, 27; Blanchette et al. in Genome Inform 8:25–34, 28), and the other based on maximum parsimony phylogeny inference. We further show how to extend these heuristics to obtain solutions to the DSMT problem, that models large scale duplication events.

**Results:**

Experimental results from both simulated and real tumor data show that our methods outperform previous heuristics (Chowdhury et al. in Bioinformatics 29(13):189–98, 23; Chowdhury et al. in PLoS Comput Biol 10(7):1003740, 24) in obtaining solutions to both RSMT and DSMT problems.

**Conclusion:**

The methods introduced here are able to provide more parsimony phylogenies compared to earlier ones which are consider better choices.

## Background

Cancer is recognized to be an evolutionary process driven by mutations in tumor cells [1]. These evolutionary processes include single-nucleotide variations, insertions and deletions, copy-number aberrations, fragment duplication, structural variations and gene fusions [2]. Many experiments reveal considerable intra-tumor and inter-tumor heterogeneity [3], attributed to these evolutionary processes. Clinical implications of this heterogeneity, for example in drug resistance and disease diagnosis, have been well studied [3, 4].

Rapid, simultaneous linear and branching evolution in multiple subclones of cancer cells can be modeled by a phylogenetic tree [5]. Inferring such phylogenies facilitates the study of cancer initiation, progression, treatment, and resistance [6]. They can help pinpoint important changes that lead to the recurrence of some genome aberrations [7]. Phylogeny studies also aid in identifying genes crucial for evolution and hence may contribute to developing better cancer treatment [8–11].

Mutation patterns in cancer are characterized by frequent and widespread gains and losses of genomic material which is markedly different from what is observed in species or population level evolution [6]. The gene copy number variation is due to failures in DNA repair mechanisms (e.g., translesion synthesis and non-homologous end joining) especially during tumor development [12–15]. Gene copy number changes affect a larger fraction of the genome in cancers than do any other type of somatic genetic alteration [16, 17]. Another characteristic feature of tumor evolution is the high genetic heterogeneity found. Previous phylogenetic models for cancer [9, 18–22], either do not account for these unique characteristics of cancer evolution or are not scalable and hence of limited practical use. Thus there is need for development of new phylogenetic models with scalable algorithms that can adequately model cancer evolution. A step towards a scalable model for inferring tumor phylogeny by copy number variation was taken by Chowdhury et al. [23, 24] using FISH data.

Fluorescence in situ hybridization (FISH) was developed by bio-medical researchers in the early 1980s and has been used to detect and localize the presence or absence of specific DNA sequences and to visualize the genomic diversity of chromosome aberrations [25]. While single cell sequencing (SCS) technique also has the potential to count the number of specific genes or specific regions for a group of cells, the highly non-uniform coverage, the admixture signal and relatively high cost make the current SCS technique unsuitable. By allowing us to count copies of gene probes across hundreds to thousands of cells, FISH provides a way to characterize tumor heterogeneity reliably.

Chowdhury et al. [23] model the progression of tumor cells from the FISH copy number data and show that such a progression of FISH cell count patterns over a tree effectively models the evolution of tumor cells. They assume a parsimonious model describing evolution by single gene copy number changes [23] and later extend it to incorporate large scale duplication events (including chromosomal and whole genome duplication events) [24]. They reduce the modeling problem to the NP-hard rectilinear Steiner minimum tree (RSMT) problem and a more general duplication Steiner minimum tree (DSMT) problem, and develop heuristics to construct RSMT/DSMT trees. RSMT/DSMT topologies and other tree-based statistics yield insights into selective pressure which simpler statistics (like cell counts) do not and provide independent support to clinical findings such as in [26]. They also are useful as discriminatory features in down-stream classification-based analyses. Earlier experiments [23, 24] suggest that better phylogeny inference models can potentially improve these analyses that rely on accurate RSMT/DSMT inference.

A model based on the Steiner minimum tree has also been introduced in the “small phylogeny” problem at both the sequence level [27] and the gene order level [28]. Given a phylogenetic tree structure and genomes (sequences or gene orders) at the leaf vertices, the “small phylogeny” problem attempts to reconstruct all the ancestral genomes at internal vertices such that the total number of evolutionary operations, measured by the sum of distances between adjacent genomes, is minimized. A special case of the “small phylogeny” problem is called the median problem—given three genomes, find the configuration of a median genome to minimize the sum of the pairwise distances between the median and three input genomes [29]. Sankoff et al. propose methods to find approximate solutions that iteratively solve the median problem for one internal vertex at a time until a local optimum to the Steiner minimum tree is found [27, 28].

Since FISH [23] yields cell count patterns of gene copy numbers at single-cell resolution, parsimony-based phylogenetic approaches (designed previously for building phylogenies of species) can be applied to such data. Maximum parsimony approaches seek the tree and the cell count patterns (gene copy numbers) for the internal nodes that minimize the total number of events needed to produce the given input from a common ancestor. Although this also results in an NP hard formulation, several heuristics have been developed in the last decade to solve the Maximum Parsimony Phylogeny problem [30]. Packages such as TNT [31] have largely overcome computational limitations and allow reconstructions of large trees, inferring accurate trees with hundreds of taxa within minutes, and the use of continuous characters [32].

In this paper, we propose two approaches to solve the RSMT problem, one approach through iteratively optimizing the median version of RSMT problem and the other approach based on Maximum Parsimony tree reconstruction. We further show how to use heuristics developed for RMST to find approximate solutions for the DSMT problem.

Experimental results from both simulated and real tumor data show that our approaches outperform previous heuristics by finding better solutions for both RSMT and DSMT problems and thus enabling us to obtain good models for cancer phylogenies using cell count patterns from FISH data.

## Methods

In this section we describe the rectilinear Steiner minimum tree (RSMT) and the duplication Steiner minimum tree (DSMT) problems for modeling the progression of FISH cell count patterns and compare them with minimum spanning tree (MST) and maximum parsimony tree (MPT) problems. We then describe two new heuristics for obtaining approximate solutions to RSMT from MST and MPT, and show how to extend these heuristics for RSMT to obtain solutions for DSMT.

### RSMT, MST, MPT and DSMT

The rectilinear Steiner minimum tree (RSMT) problem for gene copy number changes is defined as follows [23].

Definition: RSMT(*n, d*)

Input: FISH data of *n* cell count patterns on *d* gene probes for a given patient

Output: A minimum weight tree with the rectilinear metric (or *L*_1_ distance) including all the observed *n* cell count patterns and, as needed, unobserved Steiner nodes along with their cell count patterns for *d* probes, Steiner nodes are used to represent missing nodes during process of gene copy number changes.

Each cell has some non-negative integer count of each gene probe. Given two cell count patterns (*x*_1_*, x*_2_*,..., x*_*d*_) and (*y*_1_*, y*_2_*,..., y*_*d*_), the pairwise distance under the rectilinear metric (or *L*_1_ distance) is defined as $\left| {x_{1} - y_{1} } \right| + |x_{2} - y_{2} | + \cdots + |x_{d} - y_{d} |$, where $x_{i} ,y_{i} \in {\text{N}}$*x*_*i*_*, y*_*i*_ N*****. The weight of a tree with nodes labeled by cell count patterns is defined as the sum of all branch lengths under the rectilinear metric. Since the distance between two cell count patterns under the rectilinear metric represents the number of single gene duplication and loss events between them, a minimum weight tree, including Steiner nodes if needed, explains the *n* observed cell count patterns of *d* probes with minimum total number of single gene duplication and loss events, from a single ancestor. The single ancestor could be, for example, cell count pattern with a copy number count of 2 for each gene probe (a healthy diploid cell) [23, 24]. The RSMT problem is NP-complete [33].

If all possible cell count patterns in cancer cells are present as the input, then the RSMT is simply the MST, since no additional Steiner nodes are needed. The MST problem for gene copy number changes is defined as follows.

Definition: MST(*n, d*)

Input: FISH data of *n* cell count patterns on *d* gene probes for a given patient

Output: A minimum weight tree with the rectilinear metric (or *L*_1_ distance) including all the observed *n* cell count patterns.

Since both the minimum spanning tree and the minimum spanning network can be constructed efficiently, previous heuristics have approximated RSMT by adding additional Steiner nodes to the minimum spanning network [23, 24].

If all possible cell count patterns in cancer cells are considered to be all the *n* leaf nodes of a tree, then the RSMT problem becomes the MPT problem, since a MPT can be viewed as a Steiner tree of *n* leaf nodes and (n − 2) additional internal/Steiner nodes. The maximum parsimony tree problem for phylogenetic inference of gene copy number changes is defined as follows.

Definition: MPT(*n, d*)

Input: FISH data of *n* cell count patterns on *d* gene probes for a given patient

Output: A minimum weight unrooted binary tree with the rectilinear metric (or *L*_1_ distance) including all the observed *n* cell count patterns as leaves and *n* − 2 unobserved internal nodes

The MPT problem is also NP complete [34] but heuristics like TNT [31], have largely overcome computational limitations and allow reconstructions of large trees and the use of continuous characters [32]. The copy number of each gene can be treated as continuous characters and TNT can be used to find the minimum weight phylogenetic tree.

The above problem definitions use the rectilinear metric to model single gene duplication and loss events. Chowdhury et al. [24] generalize the distance metric to incorporate large scale duplication events including chromosomal duplication and whole genome duplication. The duplication Steiner minimum tree (DSMT) problem is defined as follows.

Definition: DSMT(*n, d*)

Input: FISH data of *n* cell count patterns on *d* gene probes for a given patient

Output: A minimum weight tree with a generalized metric [24] (incorporating large scale duplication events) including all the observed *n* cell count patterns and, as needed, unobserved Steiner nodes along with their cell count patterns for *d* probes, Steiner nodes here are used to represent missing nodes during the process of gene copy number changes.

### From MST to RSMT

The median version of the RSMT problem can be solved in linear time.

#### **Theorem 1**

*RSMT(3, d) can be solved in time O(d).*

*Proof* Given three cell count patterns $(X_{1}^{1} ,X_{2}^{1} , \ldots ,X_{n}^{1} )$, $(X_{1}^{2} ,X_{2}^{2} , \ldots ,X_{n}^{2} )$ and $(X_{1}^{3} ,X_{2}^{3} , \ldots ,X_{n}^{3} )$, RSMT(3*, d*) returns a cell count pattern (*m*_*1*_*, m*_*2*_*,...,m*_*d*_) such that $\sum\nolimits_{i = 1}^{3} {\sum\nolimits_{j = 1}^{d} {|X_{j}^{i} - m_{j} |} }$ is minimized, where $X_{j}^{i} ,m_{j} \in N$. Since the count for each gene probe is independent, we can optimize *mj* independently which minimizes $\sum\nolimits_{i = 1}^{3} {\left| {X_{j}^{i} - m_{j} } \right|}$, respectively, and *mj* simply equals to the median of $X_{j}^{1} ,X_{j}^{2} {\text{ and }}X_{j}^{3} .$ Thus (*m*1, *m*2, …, *md*) can be constructed in time *O*(*d*) and if it differs from all three input cell count patterns then a Steiner node with cell count pattern (*m*1*, m*2*,…, md*) has to be introduced. On the other hand, $\sum\nolimits_{j = 1} {\min_{y \in N} } \sum\nolimits_{i = 1}^{3} {|X_{j}^{i} - y|}$ is a lower bound for the minimum weight of any Steiner tree on three input cell count patterns, and $\arg \min_{y \in N} \sum\nolimits_{i = 1}^{3} {|X_{j}^{i} - y|} = m_{j}$, thus the above construction is optimal under the rectilinear metric.

**Fig. 1:**
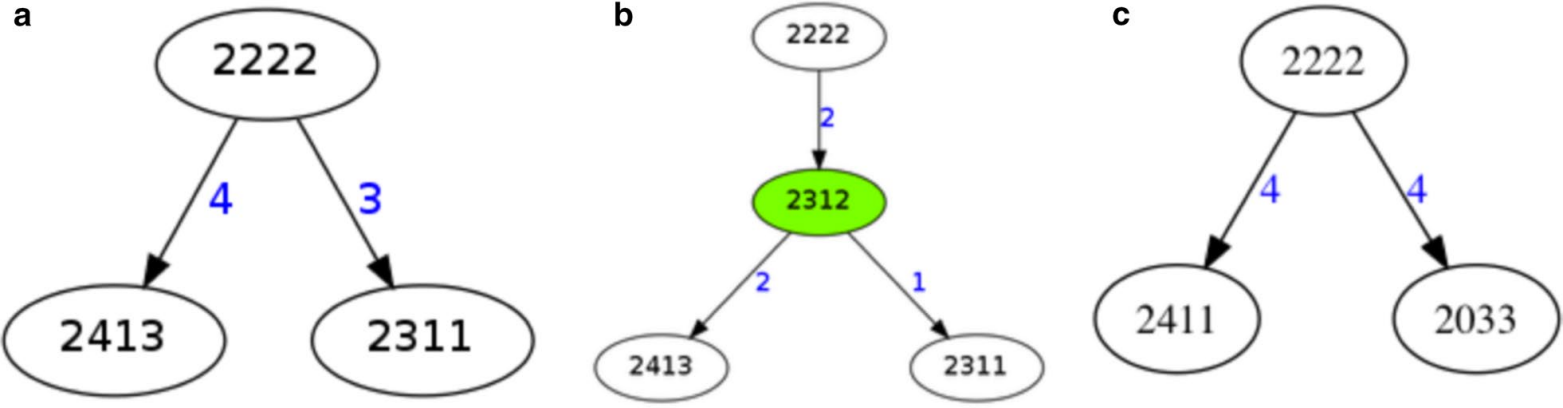
Instances of RSMT(3, d) and the introduction of the Steiner node as the median. **a** shows an example of triple original nodes. **b** shows a case where a steiner node can be added to the original triple original nodes. **c** shows one example where no steiner node can be added

Two instances of RSMT(3, d) are shown in Fig. 1(a, b, c). Given three cell count patterns in Fig. 1(a), a Steiner node is introduced in Fig. 1(b) which reduces the weight of the tree (i.e., the number of single gene duplication and loss events) from 7 to 4. Figure 1(c) shows an instance where no Steiner node is introduced.

Sankoff et al. study iterative methods to find approximate solutions to the Steiner tree problem. They solve the median problem for one internal vertex at a time, iteratively improving the solution until a local optimum is found [27, 28]. For each internal node in the (binary) tree, in each iteration, the input for a median instance consists of its three immediate neighbors [28].

**Fig. 2:**
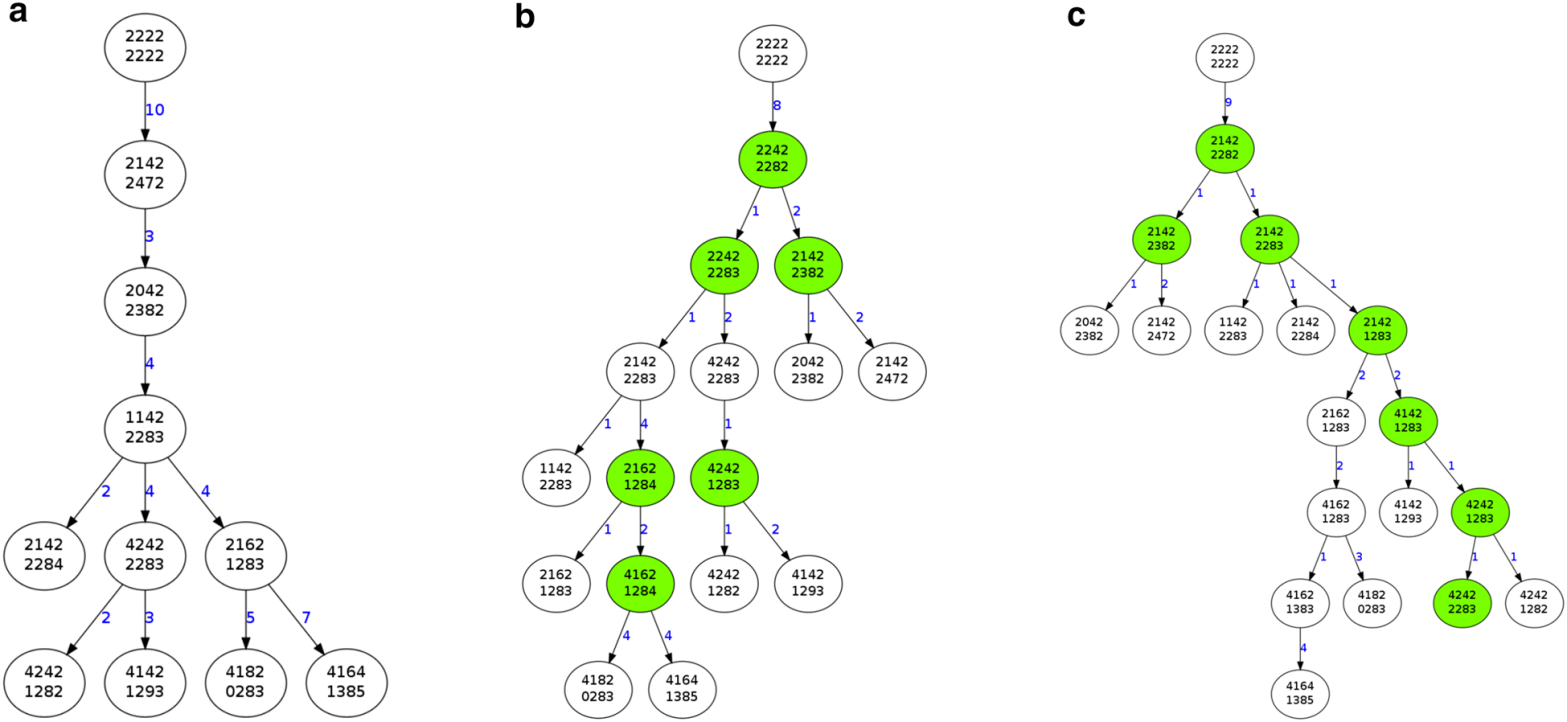
Different orders of adding Steiner nodes result in different weights of the resulting trees. *White nodes* means original cell patterns while *green nodes* stand for steiner nodes. **a**, **b**, **c** share the same original cell patterns. **a** is one minimum spanning tree without introducing any steiner nodes. The steiner node (21422282) is introduced first for **b** and last for **c**

Our algorithm is based on the observation that the order in which Steiner nodes are added to a tree affects the final weight of the resulting tree. For example, Fig. 2(a) shows the original tree before iterative optimization, and Fig. 2(b, c) show two different orders in which Steiner node (21422282) is introduced resulting in different tree scores.

**Fig. 3:**
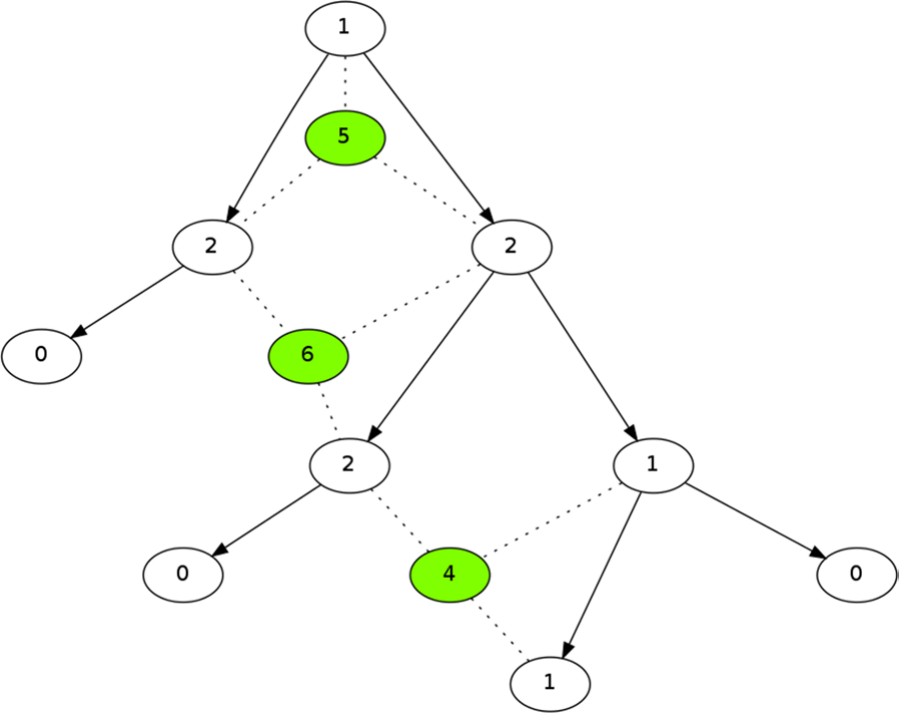
The definition of *Steiner count* of the existing nodes and the *inference score* of potential Steiner nodes to be added

We define the *Steiner count* of any node to be the number of triplets which contain the node and require the introduction of a Steiner node to optimize the tree weight. The *inference score* for each potential Steiner node with respect to a triplet is thus defined as the sum of *Steiner counts* of the three nodes in that triplet. At each iteration of our algorithm, the potential Steiner node with minimum *inference score* is added to minimize the inference score from other potential Steiner nodes with respect to the current tree. An example is shown in Fig. 3.

Our iterative algorithm starts from a MST built from the set of input cell count patterns, selects a median instance at a time, and iteratively improves the solution until a local optimum is found. The detailed description is given in Algorithm 1.



### From MPT to RSMT

**Fig. 4:**
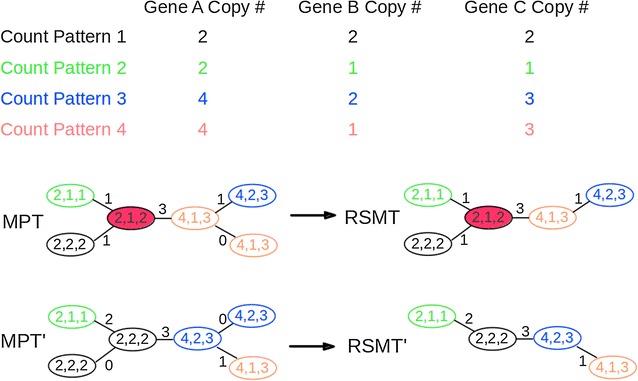
*Top* The input data of 4 cell count patterns on 3 genes. *Bottom* Two maximum parsimony trees MPT and MPT′, both of weight 6, are shown on the *left*. Nodes with identical cell count patterns are shown in the *same color* in both MPT and MPT′. The corresponding RSMT and RSMT′, both of weight 6, are shown on the *right*, and the Steiner node in RSMT is colored in *red*

In general, there may be multiple optimal solutions for the MPT problem, e.g., the internal nodes labeled by different cell count patterns. In any MPT with all nodes labeled by cell count patterns, a branch is called *trivial* if its length is 0 under the rectilinear metric. For any MPT, an unobserved internal node is a Steiner node if and only if it is labeled by a distinct cell count pattern other than any input cell count patterns. If we contract all trivial branches in MPT, the remaining unobserved internal nodes will be the Steiner nodes in RSMT. See Fig. 4 for an example.

#### Minimizing Steiner nodes

The MPT, as obtained above, may contain up to (*n* − 2) Steiner nodes. Following the philosophy of parsimony, we seek to minimize  these artificially introduced nodes, although this step does not reduce the final tree weight and is not required by the formal definition of RSMT (which does not place any explicit constraints on the number of Steiner nodes). In fact, all the previous heuristics [23, 24, 35] also implicitly do not add unnecessary Steiner nodes and thus are biased towards a parsimonious solution due to their incremental way of adding Steiner nodes to an initial tree with no Steiner nodes.

**Fig. 5:**
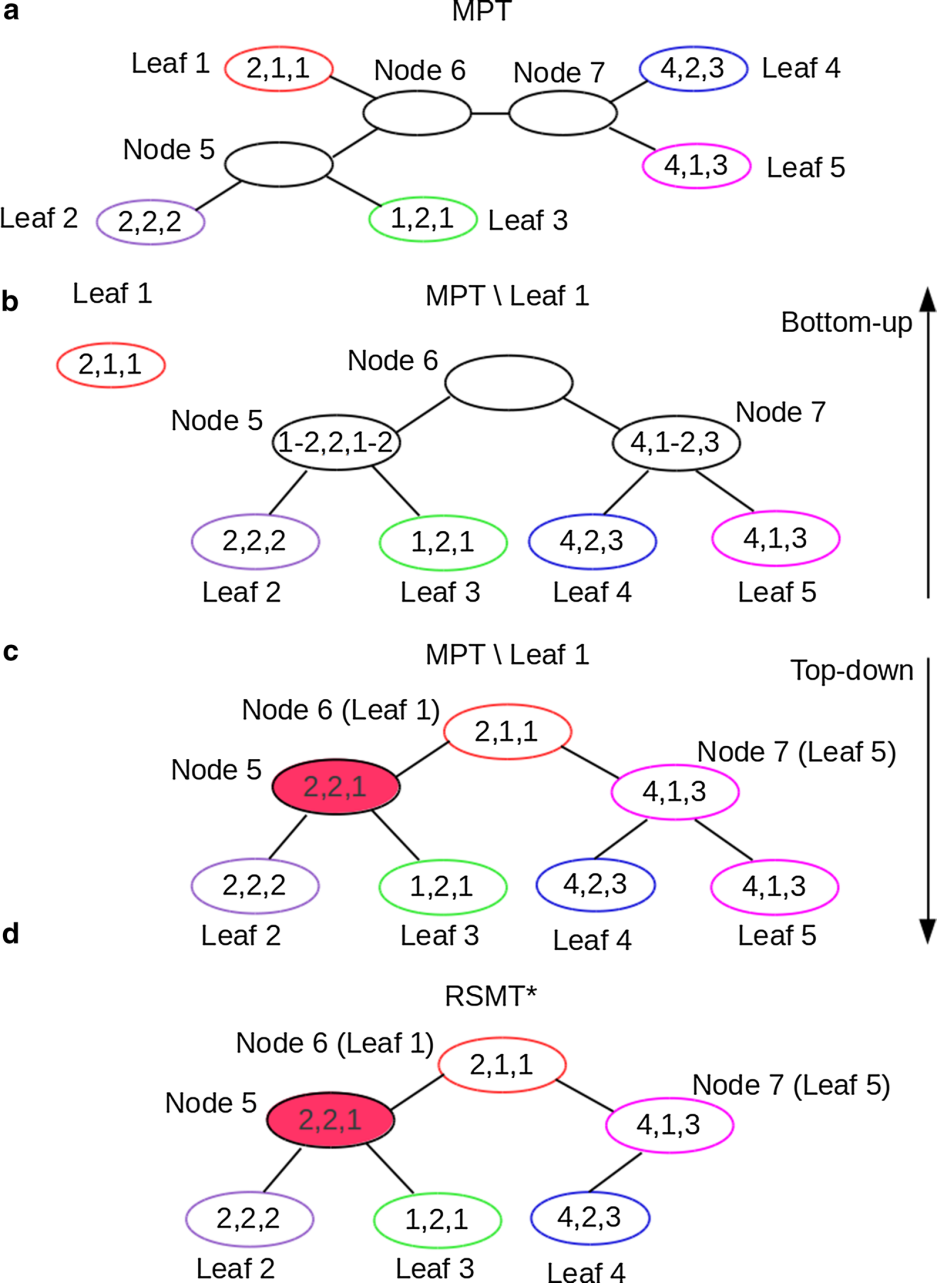
An example to test whether *Leaf*1 can be optimally “lifted” to its parent node *Node*6 in MPT. **a** A MPT on 5 leaves and 3 internal nodes. **b** *Leaf*1 and compute the ranges of possible values to internal nodes, except *Node*6, in *MPT Leaf*1 in a* bottom-up* phase. **c** Assign the cell count pattern of *Leaf*1 to the root of *MP T Leaf*1, and determine the values for other internal nodes in *MPT Leaf*1 in a* top-down* phase. **d** Contract all trivial branches in *MPT Leafi* and derive RSMT*. Nodes with identical cell count patterns are shown in the* same color* and the Steiner node in RSMT* is colored in *red*

Given any MPT, if the internal nodes are labeled by cell count patterns, the RSMT can be derived by contracting all its trivial edges; but the MPT obtained does not have labels assigned to the internal nodes. Hence the problem reduces to finding the best possible labels for internal nodes that does not increase the weight. The dynamic programming (DP) method of [36] can be adapted to find the internal labels, but modifications are needed to account for the rectilinear metric and its implications on the total tree weight. Our algorithm proceeds by finding whether a leaf label can be reused in (or “lifted” to) its parent for each leaf in the tree. If a leaf can be “lifted” to its parent, the leaf is removed from the tree and its parent is chosen to be the root. In the bottom–up phase of the DP, labels from all other leaves are propagated up the tree by using ranges of cell count patterns that can maintain the leaf cell counts without increasing the tree weight. In the top–down phase, cell count values are assigned to the internal nodes and a candidate tree is generated by contracting trivial edges. Several such candidate trees are generated by selecting different root nodes from lifted leaves. We choose a candidate tree with minimum number of Steiner nodes, with no increase in tree weight. The complete algorithm is presented in Algorithm 2 and a detailed example is shown in Fig. 5.

### From RSMT to DSMT

Cancer genomes are prone to large scale duplications (including duplication of the entire chromosome or genome), but the above two heuristics for RSMT only take into account single gene duplication and loss events and thus may be of limited biological interest. In the following, we show how to extend the heuristics for RSMT to derive approximate solutions for DSMT.

We follow the idea from Chowdhury et al. [24] to first identify possible large scale duplications. Specifically, given a tree reconstructed by [24] for DSMT, we first locate all branches containing large scale duplications (including both chromosomal and whole genome duplications). We then remove such branches, and thus split the tree into disjoint subtrees. For each subtree, we use only the leaf genomes as the input and reconstruct a new RSMT tree by using the above two heuristics (described in “From MST to RSMT” and “From MPT to RSMT” sections). Finally, we re-insert the removed branches and thus assemble the reconstructed RSMT subtrees into a new tree which is our approximate solution for DSMT.

## Experimental results

In the following, we refer to previous heuristics as FISHtree [23, 24],^1^ our MST-based iterative approach MSTtree, and our MPT-based approach as MPTtree. We also refer to the exact method [23] as EXACTtree.

### Real cancer datasets

We use both the real cervical cancer and breast cancer data samples and simulation samples generated through the process described by Chowdhury et al. [23, 24]. The cervical cancer data contains four gene probes LAMP3, PROX1, PRKAA1 and CCND1, and the breast cancer data contains eight gene probes COX-2, MYC, CCND1, HER-2, ZNF217, DBC2, CDH1 and p53. These genes are chosen because they are considered as important factors for cancer growth inhibition or promotion. The cervical cancer data is from 16 lymph positive patients (both primary and metastatic tumors) and 15 lymph negative patients, making 47 samples in total. The breast cancer data is from 12 patients with both IDC and DCIS and 1 patient with only DCIS, making 25 samples in total. More details of this FISH data set can be found in Chowdhury et al. [23, 24].

**Fig. 6:**
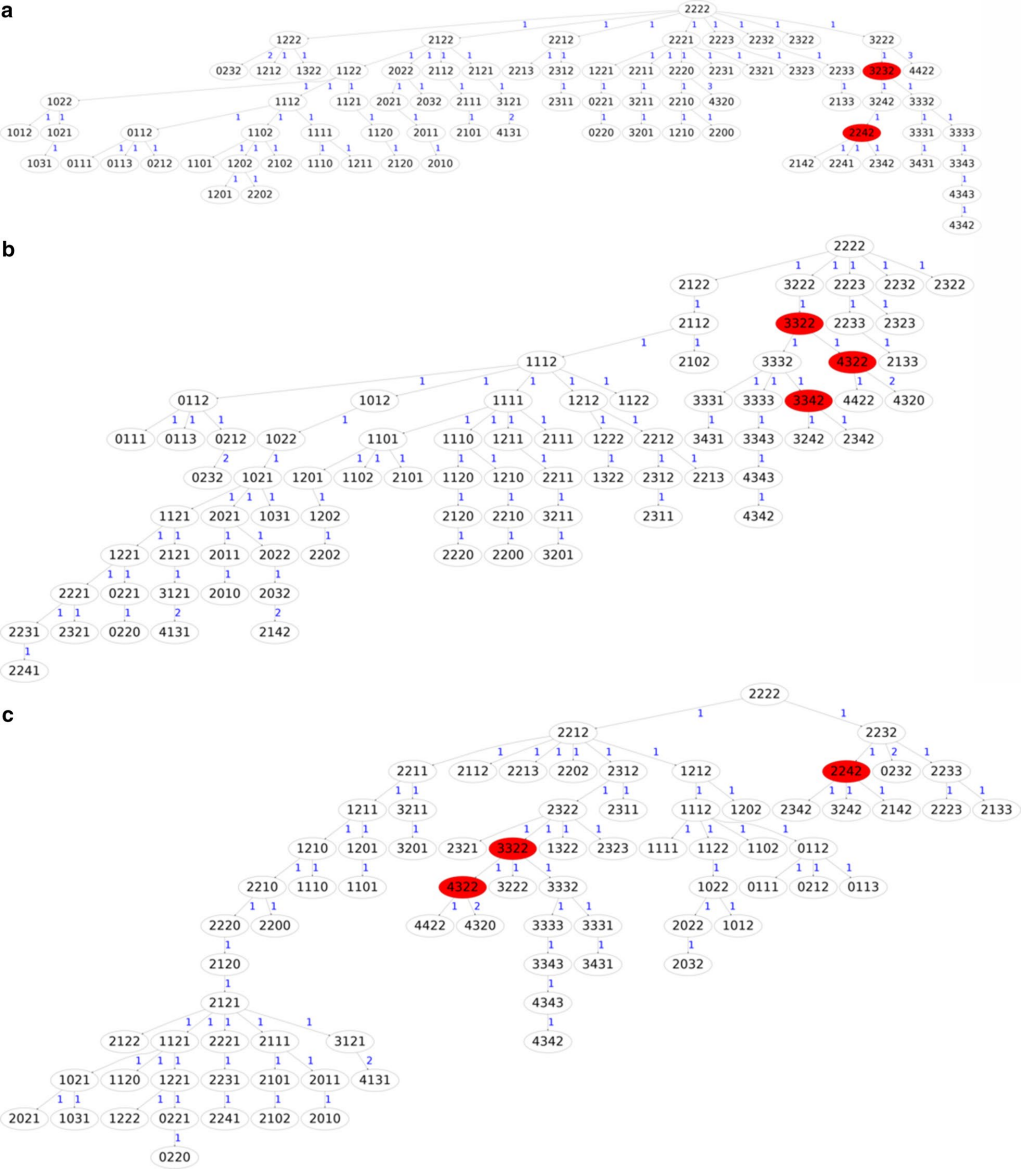
Given the metastatic cervical cancer sample of patient 12, **a** approximate RSMT constructed by FISHtree with weight 83, **b** approximate RSMT constructed by iFISHtree with weight 82 and **c** approximate RSMT constructed by mpFISHtree with weight 81. Each node in the tree is labeled by a cell count pattern of four gene probes LAMP3, PROX1, PRKAA1 and CCND1. Each *white* node represents an input cell count pattern, and each *red* node represents an inferred Steiner node. Branch lengths are shown in *blue*

For the RSMT problem, Tables 1 and 2 summarize the comparison of FISHtree [23], MSTtree and MPTtree for breast cancer samples and cervical cancer samples, respectively (best tree weights are shown in italic). Note that MPTtree performs the best in all the samples. Figure 6 shows three approximate RSMT trees for the cervical cancer sample of patient 29, constructed by FISHtree (Fig. 6(a), tree weight = 83), iFISHtree (Fig. 6(b), tree weight = 82) and mpFISHtree (Fig. 6(c), tree weight = 81), respectively.

**Table 1:** Comparison on the real datasets for RSMT on breast cancer samples. (EXACTtree results are not available due to the time limitation)

Case #	RSMT tree weight (# Steiner nodes)		
	FISHtree	MSTtree	MPTtree
B1 IDC	213 (15)	212 (13)	*211* (19)
B1 DCIS	241 (14)	242 (15)	*239* (22)
B2 IDC	217 (15)	216 (20)	*211* (22)
B2 DCIS	56 (2)	56 (2)	*55* (3)
B3 DCIS	100 (7)	*98* (7)	*98* (10)
B4 IDC	214 (16)	*213* (17)	*213* (17)
B6 IDC	112 (4)	*111* (4)	*111* (6)
B7 IDC	116 (8)	*113* (12)	*113* (12)
B7 DCIS	186 (13)	184 (14)	*182* (22)
B9 IDC	222 (22)	217 (25)	*213* (30)
B9 DCIS	164 (12)	163 (13)	*161* (15)
B10 IDC	128 (4)	128 (4)	*127* (4)
B10 DCIS	146 (6)	*145* (8)	*145* (9)
B11 DCIS	136 (6)	135 (7)	*134* (7)
B12 IDC	201 (9)	200 (10)	*198* (15)
B12 DCIS	161 (9)	161 (10)	*158* (13)
B13 IDC	132 (7)	*131* (8)	*131* (8)
B13 DCIS	63 (3)	*62* (4)	*62* (4)

The best tree weights are shown in italics for each sample. The number of Steiner nodes is shown in parenthesis. Seven breast cancer samples have ties in tree weights and thus are not included due to the space limit

**Table 2:** Comparison on the real datasets for RSMT on cervical cancer samples

Case #	RSMT tree weight (# Steiner nodes)			
	FISHtree	MSTtree	MPTtree	EXACTtree
C5	195 (13)	196 (12)	*194* (13)	*194* (13)
C6	82 (2)	82 (2)	*81* (5)	*81* (4)
C8	103 (6)	103 (6)	*100* (9)	*100* (8)
C9	143 (1)	*142* (2)	*142* (5)	*142* (2)
C10	87 (0)	*86* (1)	*86* (1)	*86* (1)
C12	72 (1)	*71* (2)	*71* (2)	*71* (2)
C13	150 (5)	150 (5)	*149* (7)	*149* (7)
C15	74 (1)	*73* (2)	*73* (2)	*73* (2)
C18	127 (4)	127 (4)	*126* (6)	*126* (6)
C21	*73* (4)	74 (3)	*73* (5)	*73* (4)
C27	59 (1)	*57* (3)	*57* (2)	*57* (3)
C29	83 (2)	82 (3)	*81* (3)	*81* (3)
C30	118 (9)	118 (9)	*116* (9)	*116* (10)
C32	209 (7)	207 (9)	*205* (14)	*205* (13)
C34	83 (5)	*82* (6)	*82* (6)	*82* (6)
C35	67 (1)	67 (1)	*66* (2)	*66* (3)
C42	199 (7)	198 (9)	*197* (12)	*197* (11)
C45	172 (10)	*169* (13)	*169* (14)	*169* (15)
C46	110 (5)	109 (6)	*108* (8)	*108* (7)
C49	162 (4)	*161* (5)	*161* (7)	*161* (7)
C53	80 (3)	*79* (4)	*79* (4)	*79* (4)
C54	146 (6)	145 (7)	*144* (10)	*144* (9)

The best tree weights are shown in italics for each sample. The number of Steiner nodes is shown in parenthesis. 24 cervical cancer samples have ties in tree weights and thus are not included due to the space limit

For the DSMT problem, we compare FISHtree [24] and MPTtree, since MPTtree outperforms MSTtree for RSMT. We summarize the results on breast cancer samples and cervical cancer samples in Tables 3 and 4 (better tree weights are shown in italic). Similarly, MPTtree outperforms FISHtree in both breast cancer samples and cervical cancer samples.

**Table 3:** Comparison on the real datasets for DSMT on breast cancer samples: number of times and percentage that the best scoring tree (including ties) is obtained by FISHtree and MPTtree

Cell line	DSMT best score	
	FISHtree	MPTtree
B1 IDC	217	*206*
B1 DCIS	150	*140*
B2 IDC	203	*189*
B3 DCIS	99	*97*
B4 IDC	203	*193*
B5 IDC	64	*63*
B6 IDC	108	*106*
B6 DCIS	*42*	43
B7 IDC	116	*115*
B10 IDC	125	*123*
B11 DCIS	122	*121*
B12 IDC	125	*123*
B12 DCIS	162	*149*
B13 IDC	132	*129*
B13 DCIS	63	*61*

Italic font is used for the cases with lower weights

**Table 4:** Comparison on the real datasets for DSMT on cervical cancer samples: number of times and percentage that the best scoring tree (including ties) is obtained by FISHtree and MPTtree

Cell Line	DSMT Best score	
	FISHtree	MPTtree
C6	82	*81*
C8	95	*93*
C18	126	*122*
C24	*201*	204
C29	80	*76*
C34	*81*	82
C53	75	*71*

Italic font is used for the cases with lower weight

Note that both the RSMT and DSMT problems are NP-hard and so obtaining optimal solutions can be very difficult. Although the improvements in terms of tree weights appear small, coming closer to the optimal tree even by a few units is challenging. The improvements are more clearly seen on simulated data in the following section.

### Simulated cancer data

We test on simulated datasets generated for different number of gene probes (4, 6, 8) and for different tree growth factors (0.4 and 0.5) [23, 24]. For each pair of parameters, we simulate 200 samples with the number of distinct cell count patterns varying from 120 to 150.

For the RSMT problem, Table 5 summarizes the number of times each of the methods, FISHtree, MSTtree, MPTtree and EXACTtree, obtains the best results on these simulation datasets. For the DSMT problem, Table 6 summarizes the number of times each of the methods, FISHtree and MPTtree, obtains the better results on these simulation datasets.

**Table 5:** Comparison on simulated datasets for RSMT: number of times and percentage that the best scoring tree (including ties) is obtained by the four methods

Probe #	Growth factor	RSMT Best score count		(Best score percentage)		
		FISHtree	MSTtree	MPTtree		EXACTtree
4	0.4	92 (46 %)	137 (68.5 %)	196 (98 %)		200
6	0.4	70 (35 %)	98 (49 %)	194 (97 %)		N/A
8	0.4	41 (20.5 %)	69 (34.5 %)	196 (98 %)		N/A
16	0.4	N/A	53 (26.5 %)	200 (100 %)		N/A
4	0.5	93 (46.5 %)	130 (65 %)	194 (97 %)		200
6	0.5	68 (34 %)	99 (49.5 %)	196 (98 %)		N/A
8	0.5	40 (20 %)	64 (32 %)	195 (97.5 %)		N/A
16	0.5	N/A	57 (28.5 %)	200 (100 %)		N/A

EXACTtree results for datasets with over four gene probes are not available due to the time limitation

**Table 6:** Comparison on simulated datasets for DMST: number of times and percent- age that the best scoring tree (including ties) is obtained by FISHtree and MPTtree

Probe #	Growth factor	DMST Best score count	(Best score percentage)	
		FISHtree	MPTtree	
4	0.4	175 (87.5 %)	191 (95.5 %)	
6	0.4	145 (35 %)	194 (97 %)	
8	0.4	101 (50.5 %)	199 (99.5 %)	
4	0.5	178 (89 %)	189 (94.5 %)	
6	0.5	147 (73.5 %)	193 (96.5 %)	
8	0.5	93 (46.5 %)	200 (100 %)	

MPTtree performs the best in all the simulation datasets. Due to the very efficient implementation of TNT [37], the running time of MPTtree is comparable to that of FISHtree, MSTtree, all of which are orders of magnitude faster than the exact method (we could not obtain the optimal solutions within a reasonable amount of time when there are more than 6 gene probes—shown as N/A in Tables 1 and 5).

## Discussion

Both the RSMT and DSMT have been shown to be reasonable models for progression of cancer cells using FISH cell count pattern data [23, 24]. Efficient heuristics are necessary to obtain approximations to RSMT/DSMT since finding the optimal solution is NP-hard. We present two new algorithms to approximate RSMT, one from the MST, and the other from the MPT. We also show how to extend these heuristics for RSMT to obtain approximate solutions for DSMT. Our experiments on both synthetic and real datasets demonstrate the superiority of our algorithms over previous methods in obtaining better parsimonious models of cancer evolution.

RSMT instances found by our heuristics may have multiple solutions with the same tree weight and exploring strategies to choose the best from multiple.

RSMT solutions remains open problems. Methods to provide reliable bootstrap-based confidence scores [38, 39] for the inferred tumor phylogenies would also be worth exploring.

## Footnotes

1 We use the best result derived from the heuristic option in [23] and the option PLOIDY LESS HEURISTIC in [24] that also approximate RSMT under the case of gene copy number changes of single probes.
